# Development and external validation of models to improve prediction of osteoporosis in elderly women: interpretable machine learning

**DOI:** 10.3389/fendo.2025.1719698

**Published:** 2026-01-09

**Authors:** Tian Tang, Shiwen Wang, Shengziyi Cai, Yun Hu

**Affiliations:** 1 Department of Geriatrics, Nanjing Drum Tower Hospital, Nanjing Drum Tower Clinical College, Nanjing University of Chinese Medicine, Nanjing, China; 2 Department of Geriatrics, Nanjing Drum Tower Hospital, Nanjing Drum Tower Clinical Medical College, Nanjing Medical University, Nanjing, China

**Keywords:** diabetes mellitus, elderly women, machine learning, osteoporosis, predictive model

## Abstract

**Introduction:**

As populations age and the prevalence of osteoporosis (OP) increases, osteoporotic fractures substantially raise disability and mortality and impose growing economic burdens, threatening health and quality of life. This study aimed to develop and externally validate a reliable, practical machine learning model to predict OP in older women using routine clinical test results and comorbidity data.

**Methods:**

We retrospectively assembled an internal dataset from NHANES (2003–2020) and randomly split it 70:30 into training and test sets. An external cohort from a Chinese tertiary hospital was used for validation. Predictors were selected using LASSO in the training set. Five algorithms (XGBoost, SVM, RF, LightGBM, and Naive Bayes) were tuned, and model performance was evaluated on the test set and in the external cohort. Calibration curves and decision curve analysis (DCA) were used to assess calibration and clinical net benefit. Feature contributions were quantified with Shapley additive explanations (SHAP).

**Results:**

Among 3,950 women in the internal dataset, 833 (21.1%) had OP; in the external cohort (n=338), 167 (49.4%) had OP. SHAP ranked predictors (high to low) as: age, drinking, diabetes, eGFR, HbA1c, BMI, HDL, TG, BUN, and TBIL. After hyperparameter tuning, RF achieved an AUC of 0.805 in the internal test set and 0.740 in the external cohort; in the internal test set, accuracy was 0.82, precision 0.83, and specificity 0.97. Calibration was acceptable, and DCA indicated clinical utility across relevant thresholds.

**Conclusion:**

A random forest model using readily available clinical data predicts osteoporosis risk in older women with robust internal and external performance. The deployed model outputs calibrated probabilities at the patient level, provides case level explanations using SHAP, and supports dynamic rescoring as new routine results become available, enabling individualized risk management in routine care.

## Highlights

- A machine learning model was developed to enable early identification of osteoporosis in elderly women.
- External validation using datasets from both the United States and China demonstrated robust generalizability across populations.
- SHAP interpretation pinpoints key predictors (age, BMI, TC, HDL, HbA1C, BUN, TBIL, eGFR, DM, and alcohol consumption), supporting targeted DXA screening and early intervention in elderly women.

## 1 Introduction

Osteoporosis is a chronic metabolic disorder marked by the deterioration of bone tissue architecture and a reduction in bone mass, and is particularly common in elderly women (1). In the United States, approximately 12.6% of adults aged 50 and older are affected by osteoporosis, with a higher rate in women (19.6%) than in men (4.4%) (2). The global prevalence of osteoporosis is estimated to be 19.75% (3). Over the past three years, their burden has continued to increase. As the global population ages, the disability, mortality, and economic burden caused by osteoporosis-related fractures continue to rise, posing a serious threat to the health and quality of life of the elderly population (4). Approximately 20% to 30% of individuals with osteoporosis die within one year following an osteoporotic fracture. Since osteoporosis is typically asymptomatic prior to fracture, early screening and detection are key strategies in the management of osteoporosis.

Currently, dual-energy X-ray absorptiometry (DXA) is considered the gold standard for the diagnosis of osteoporosis, but its high cost and limited accessibility restrict its widespread use in primary care settings or among the general population (5). Traditional risk prediction tools include the International Osteoporosis Foundation’s Osteoporosis Risk One-Minute Test and the OSTA (Osteoporosis Self-assessment Tool for Asians). The One-Minute Test is quick and simple, serving as an initial screening tool for osteoporosis risk (6). OSTA has some practicality, but its predictive factors are limited, primarily relying on age and weight, with factors such as blood lipids, lifestyle, and chronic diseases not yet included (7).

The rise of the big data era has accelerated the integration of machine learning (ML) into the medical field. Compared to traditional clinical tools, AI based approaches offer the advantage of analyzing complex and interrelated features associated with osteoporosis, thereby improving accuracy. Machine learning, with its powerful modeling capabilities for nonlinear relationships, has provided new possibilities for the construction of disease prediction models (8). This study aims to develop and validate a predictive osteoporosis model in elderly women based on artificial intelligence machine learning methods, providing them with earlier and more accurate osteoporosis risk assessments.

## 2 Methods

### 2.1 Study population

According to the purpose of the survey, the data inclusion criteria for this study are as follows. Internal dataset: Data were collected from the (National Health and Nutrition Examination Survey) NHANES database for participants surveyed between 2003 and 2020, with a total of 31,306 female records collected. This study referenced the diagnostic criteria of the International Osteoporosis Foundation and the World Health Organization (4). Osteoporosis is defined as meeting the following criteria (1): bone mineral density T-score ≤ -2.5; (2) history of multiple fragility fractures, including hip fractures, lumbar spine fractures, thoracic spine fractures, etc. A total of 833 cases with a confirmed diagnosis of osteoporosis and 3,117 cases without osteoporosis were included. Exclusion criteria: 1. Age < 60 years (excluded 932 case); 2. Data missing ≥ 40% (excluded 2,150 cases); 3. Use of corticosteroids such as prednisone or oral medications for osteoporosis treatment (excluded 437 cases). 4. Lack of bone density data or osteoporosis questionnaire survey results (excluded 23,837 cases). Ultimately, 3,950 cases were included.

External dataset: Retrospective collection of medical records from 458 elderly female patients who visited the Geriatrics Department of Nanjing Drum Tower Hospital between January 2022 and December 2024. Exclusion criteria: (1) Diagnosed with Cushing’s syndrome, thyroid disease, parathyroid dysfunction, or hypogonadism (excluded 54 cases); (2) Concurrent severe chronic diseases such as rheumatoid arthritis, periodontal disease, cirrhosis, gastrointestinal diseases, malignant tumors, or severe heart failure (excluded 56 cases); (3) inability to cooperate with examinations due to mobility issues, frailty, or communication barriers (excluded 5 cases); (4) recent acute infections (excluded 5 cases). Ultimately, 338 cases were included.

### 2.2 Data extraction

#### 2.2.1 Clinical data collection

The following demographic and clinical variables were extracted: age, body mass index (BMI), white blood cell count (WBC), red blood cell count (RBC), hemoglobin (Hb), platelet count (PLT), alanine aminotransferase (ALT), aspartate aminotransferase (AST), gamma-glutamyl transferase (γ-GT), albumin (ALB), total bilirubin (TBIL), blood urea nitrogen (BUN), fasting blood glucose (FBG), glycated hemoglobin (HbA1c), total cholesterol (TC), high-density lipoprotein cholesterol (HDL-C), triglycerides (TG), triglyceride-glucose index (TyG), and estimated glomerular filtration rate (eGFR). Additionally, information on drinking (alcohol consumption) and the presence of chronic conditions such as diabetes mellitus (DM) and hypertension (HTN) was collected. The cross-cohort harmonization of variable measurement methods, unit conversions, analyzer models, and disease definitions between NHANES and the Chinese hospital dataset is summarized in **Supplementary Table S1**.

#### 2.2.2 Bone mineral density measurement

Internal dataset: BMD in NHANES 2003–2020 was assessed by DXA using three generations of Hologic fan-beam densitometers (QDR 4500A in 2003–2010, Discovery A in 2011–2018, and Horizon A in 2019–2020). External dataset: Bone density of the lumbar spine (L1–L4), total hip, and femoral neck was measured using a dual-energy X-ray absorption meter (Lunar iDXA, GE, USA).

### 2.3 Definitions and calculation formulas for relevant indicators

The definition of drinking(alcohol consumption) is at least 12 drinks in any one year, including spirits (such as whiskey or gin), beer, wine, and any other type of alcoholic beverage.

BMI: The study subjects removed their shoes, hats, and outer clothing, and their height and weight were measured. BMI = weight (kg)/height (m^2^)

TyG index (9)=ln [TG(mg/dL)×FBG(mg/dL)/2];

According to the CKD-EPI Scr formula (2009) (10), eGFR=a×(Scr/b)^c^×(0.993)^age^, a=144, b=0.7,c: -0.329 for females with Scr ≤ 0.7 mg/dL, and -1.209 for females with Scr > 0.7 mg/dL. Serum creatinine was IDMS-traceable. eGFR was calculated using the 2009 CKD-EPI creatinine equation, with the race term fixed as “non-Black” because all participants were of Han Chinese ethnicity.

### 2.4 Model construction and evaluation validation

All continuous predictors were standardized using normalization or standardization before training for algorithms that are sensitive to feature scale. Five machine learning algorithms, extreme gradient boosting (XGBoost), support vector machine (SVM), random forest (RF), Light Gradient Boosting Machine (LightGBM), and Naive Bayes were employed to develop risk prediction models for osteoporosis in elderly women. The internal dataset from NHANES was randomly divided into a training set (70%) and a testing set (30%). Model training was conducted on the training set using double-nested cross-validation, with hyperparameter tuning to optimize performance.

The final model was selected based on performance metrics evaluated on the testing set, including the receiver operating characteristic (ROC) curve. Predictive performance was further assessed using the ROC curve, precision-recall (PR) curve, calibration curves, and decision curve analysis (DCA). Six evaluation metrics were calculated: area under the ROC curve (AUC), PPV (precision), true positive rate (TPR)/Sensitivity, true negative rate (TNR)/Specificity, negative predictive value (NPV), and F1 score.

The selected model was subsequently applied to the external dataset for validation. Shapley Additive explanations (SHAP) analysis was used to interpret the model and determine the relative contribution of each predictive feature to the overall risk estimation.

### 2.5 Statistical analyses

The missing data (< 40%) were imputed using chained equations (MICE) for multivariable imputation. Continuous variables following a normal distribution are presented as mean ± standard deviation and compared using the t-test. Skewed data are reported as median and interquartile range [M (P25, P75)] and analyzed using the Wilcoxon rank-sum test. Normality was assessed via the Kolmogorov-Smirnov test. Categorical variables are expressed as counts and percentages [n (%)] and compared using the chi-square test or Fisher’s exact test. Correlation heat map of the 10 LASSO-selected predictors (Pearson/Spearman r) in **Supplementary Figure S1**. All pairwise correlations were < 0.70, indicating low multicollinearity. Feature selection was performed using the least absolute shrinkage and selection operator (LASSO). λ was chosen as the value minimizing mean cross-validated binomial deviance in 10-fold cross-validation. All analyses were conducted using RStudio (version 4.2.3) and SPSS Statistics v 27.0. A two-sided P value < 0.05 was considered statistically significant.

## 3 Results

### 3.1 Baseline characteristics

**Figure 1:**
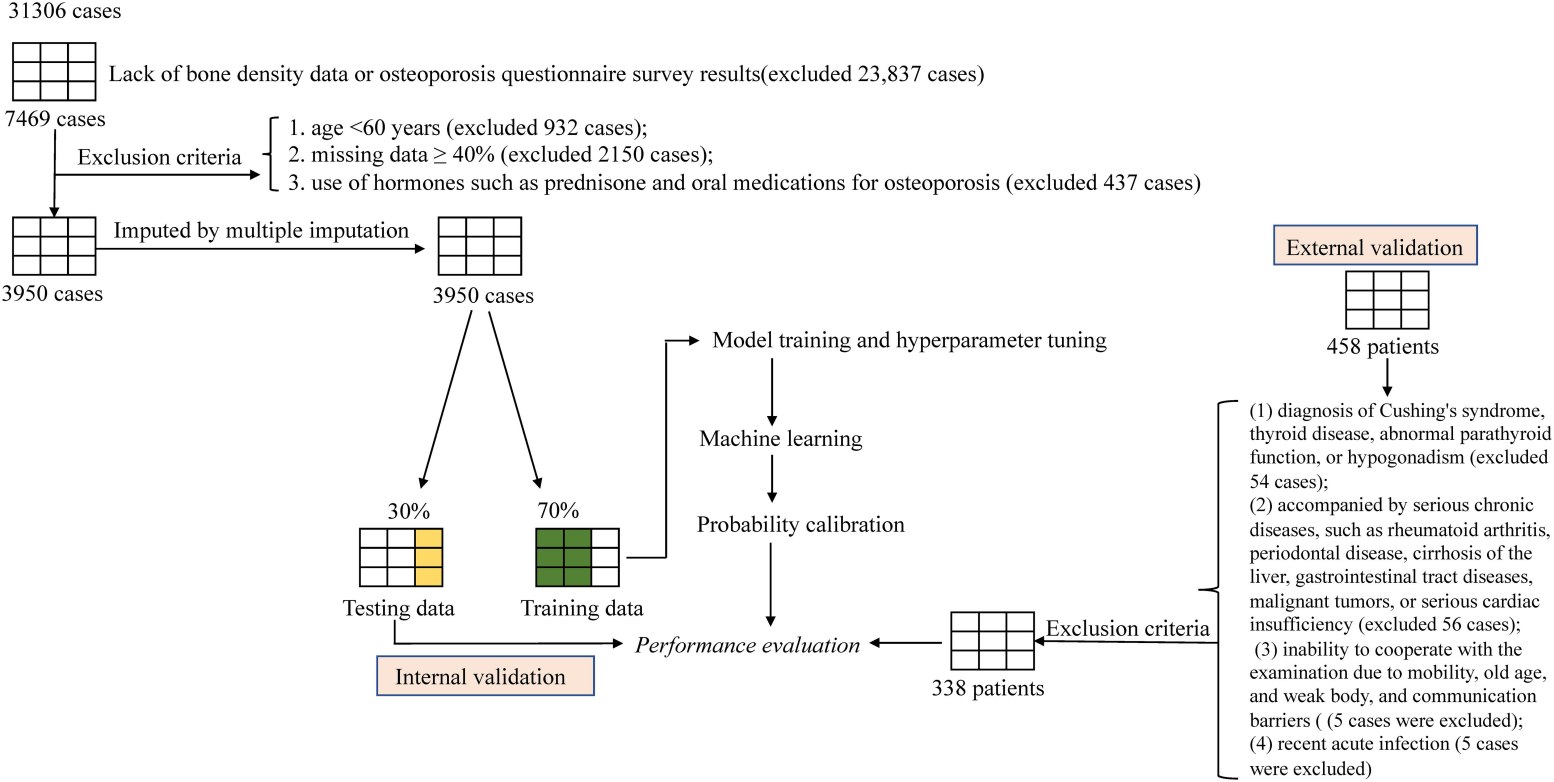
Machine learning pipeline. The flowchart outlines the study design and the steps involved in the statistical analysis.

3,950 patients from NHANES and 338 patients from Nanjing Drum Tower Hospital in China were included in further analysis. The detailed selection process of the machine learning illustrated in **Figure 1**. The internal dataset comprised 3,950 participants with a mean age of 70.26 ± 7.24 years. Among them, 833 were diagnosed with osteoporosis, corresponding to a prevalence of 21.09%. The external dataset included 338 participants with a mean age of 74.14 ± 9.67 years, among whom 167 had osteoporosis, yielding a prevalence of 49.41%. A comparison of clinical characteristics between the internal (n = 3,950) and external (n = 338) datasets is presented in **Table 1**.

**Table 1:** Comparison of clinical baseline information between the internal dataset and external dataset.

Variables	Internal dataset(n=3950)		*P* value	External dataset(n=338)		*P* value
	Non-op(n=3117)	op(n=833)		Non-op(n=171)	op(n=167)	
Age(y)	68.93 ± 6.83	75.25 ± 6.5	<0.001	71.4 ± 9.17	76.95 ± 9.38	<0.001
BMI(kg/m^2^)	28.03 ± 4.44	26.83 ± 4.6	<0.001	24.67 ± 3.45	22.83 ± 3.29	<0.001
TC(mmol/L)	5.34 ± 1.12	5.22 ± 1.06	0.005	4.63 ± 1.10	4.33 ± 1.05	0.013
HDL-C (mmol/L)	1.54 ± 0.44	1.6 ± 0.45	<0.001	1.26 ± 0.4	1.39 ± 0.47	0.006
WBC(10^9^/L)	6.6(5.5,7.9)	6.7(5.6,8.1)	0.053	5.25(4.5,6.4)	5.5(4.5,6.8)	0.272
RBC(10^12^/L)	4.44 ± 0.44	4.35 ± 0.44	<0.001	4.17 ± 0.39	4.07 ± 0.49	0.056
Hb(g/L)	134.4 ± 1.26	133.4 ± 1.21	0.027	125.56 ± 11.85	123.19 ± 12.56	0.086
PLT10^9^/L)	255.08 ± 68.43	249.57 ± 66.25	0.038	204.84 ± 59.69	195.89 ± 48.95	0.147
HbA1C(%)	6.04 ± 1.12	5.88 ± 0.86	<0.001	6.78 ± 1.67	6.54 ± 1.68	0.183
FBG(mmol/L)	6.19 ± 2.33	6.01 ± 2.09	0.034	5.77 ± 2.08	5.4 ± 2.36	0.133
ALB(g/L)	41.19 ± 3.08	40.96 ± 3.13	0.055	39.07 ± 6.59	34.98 ± 11.22	<0.001
ALT(U/L)	18 (15,23)	18(14,22)	0.002	15.8(12.6,22.1)	16(12.5,21.6)	0.993
AST(U/L)	24.22 ± 10.31	23.99 ± 7.96	0.547	20.93 ± 8.88	21.86 ± 10.41	0.375
BUN(mmol/L)	5.36(3.93,6.43)	5.71(4.28,7.5)	<0.001	5.8(4.9,7)	5.8(4.9,7.2)	0.864
γ-GT(U/L)	19(14,28)	18(14,26)	0.005	19.6(15.2,29.3)	19.6(15.1,28.6)	0.597
TBIL(umol/L)	10.26(6.84,11.97)	10.26(8.55,13.68)	0.497	9.4(7.8,12.4)	10.9(7.5,16.5)	0.013
TG(mmol/L)	1.47(1.03,2.09)	1.4(1,2.07)	0.322	1.38(0.97,1.98)	1.05(0.77,1.3)	<0.001
eGFR(ml/min/1.73m2)	72.93 ± 19.06	68.17 ± 19.51	<0.001	88.22 ± 22.27	86.13 ± 18.47	0.35
TyG	8.86 ± 0.64	8.82 ± 0.6	0.09	8.76 ± 0.74	8.32 ± 0.66	<0.001
DM, n(%)	640(20.53)	284(34.09)	<0.001	87(50.88)	76(45.51)	0.323
HTN, n(%)	1877(60.22)	521(62.55)	0.222	102(59.65)	97(58.08)	0.77
Drinking, n(%)	1180(37.86)	467(56.06)	<0.001	0(0)	1(0.59)	0.311

BMI, body mass index; TC, total cholesterol; HDL-C, high-density lipoprotein cholesterol; WBC, white blood cell count; RBC, red blood cell count; Hb, hemoglobin; PLT, platelet count; HbA1C, glycated hemoglobin; FBG, fasting blood glucose; ALB, albumin; ALT, alanine aminotransferase; AST, aspartate aminotransferase; BUN, blood urea nitrogen; γ-GT, gamma-glutamyl transferase; TBIL, total bilirubin; TG, triglycerides; eGFR, estimated glomerular filtration rate; TyG, triglyceride-glucose index; DM, diabetes mellitus; HTN, hypertension.

Statistically significant differences were observed in age, BMI, TC, HDL-C, RBC, Hb, PLT, HbA1c, FBG, ALT, BUN, γ-GT, eGFR, diabetes, and alcohol consumption (*P* < 0.05). No significant differences were found in the remaining variables (*P* > 0.05). Within the external dataset, individuals with osteoporosis exhibited significantly lower levels of age, BMI, HDL-C, TC, ALB, TBIL, TG, and TyG compared to those without osteoporosis (*P* < 0.05). Across both datasets, participants without osteoporosis consistently had higher levels of BMI, TC, RBC, Hb, PLT, HbA1c, FBG, ALB, TG, eGFR, and TyG (**Table 1**).

### 3.2 Feature selection

**Figure 2:**
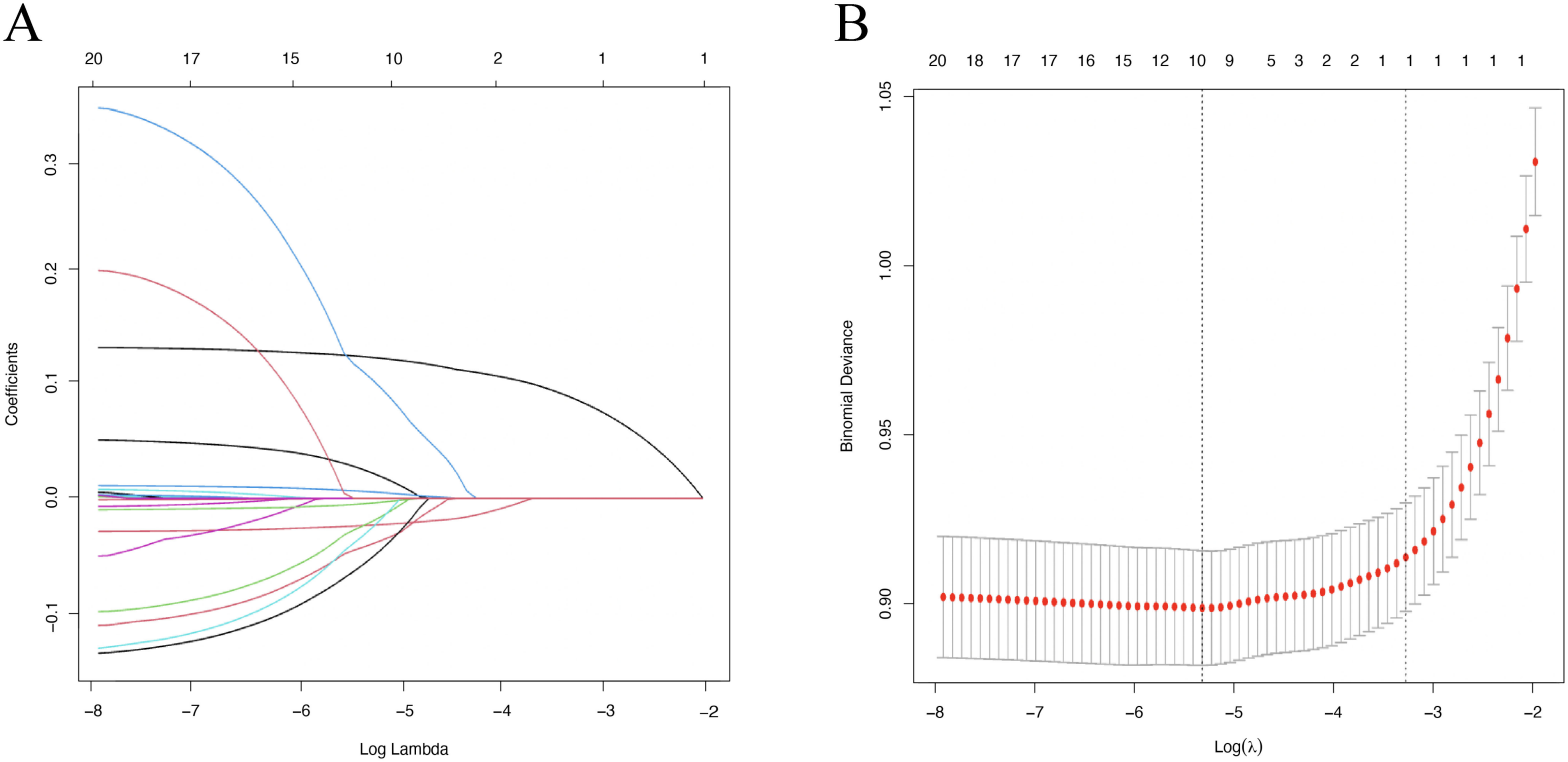
Predictor variables were selected using the least absolute shrinkage and selection operator (LASSO) regression method. **(A)** Coefficient curves were generated based on the log(lambda) sequence, and the optimal lambda value was used to identify predictors with non-zero coefficients. **(B)** The optimal lambda selection in the Lasso regression with 10-fold cross-validation.

Further analysis using LASSO regression identified ten factors significantly associated with osteoporosis in elderly women: age, BMI, TC, HDL, HbA1C, BUN, TBIL, eGFR, DM, and alcohol consumption (**Figure 2**).

### 3.3 Evaluate fairness for the NHANES cohort and the external cohort

The NHANES cohort includes Mexican Americans, other Hispanics, on-Hispanic whites, non-Hispanic blacks, and other races-Including Multi-Racial. External cohorts are all Han Chinese. In **Supplementary Table S2**, the 10 predictors derived using Lasso for each subgroup were evaluated through traditional logistic regression models, assessing AUC (95% CI), sensitivity, and specificity. Overall, discrimination was similar across subgroups. The area under the ROC curve (AUC) ranged from 0.713 to 0.777 in all groups, with overlapping 95% confidence intervals, and no subgroup showed a marked loss of performance.

### 3.4 Model construction and comparison

**Figure 3:**
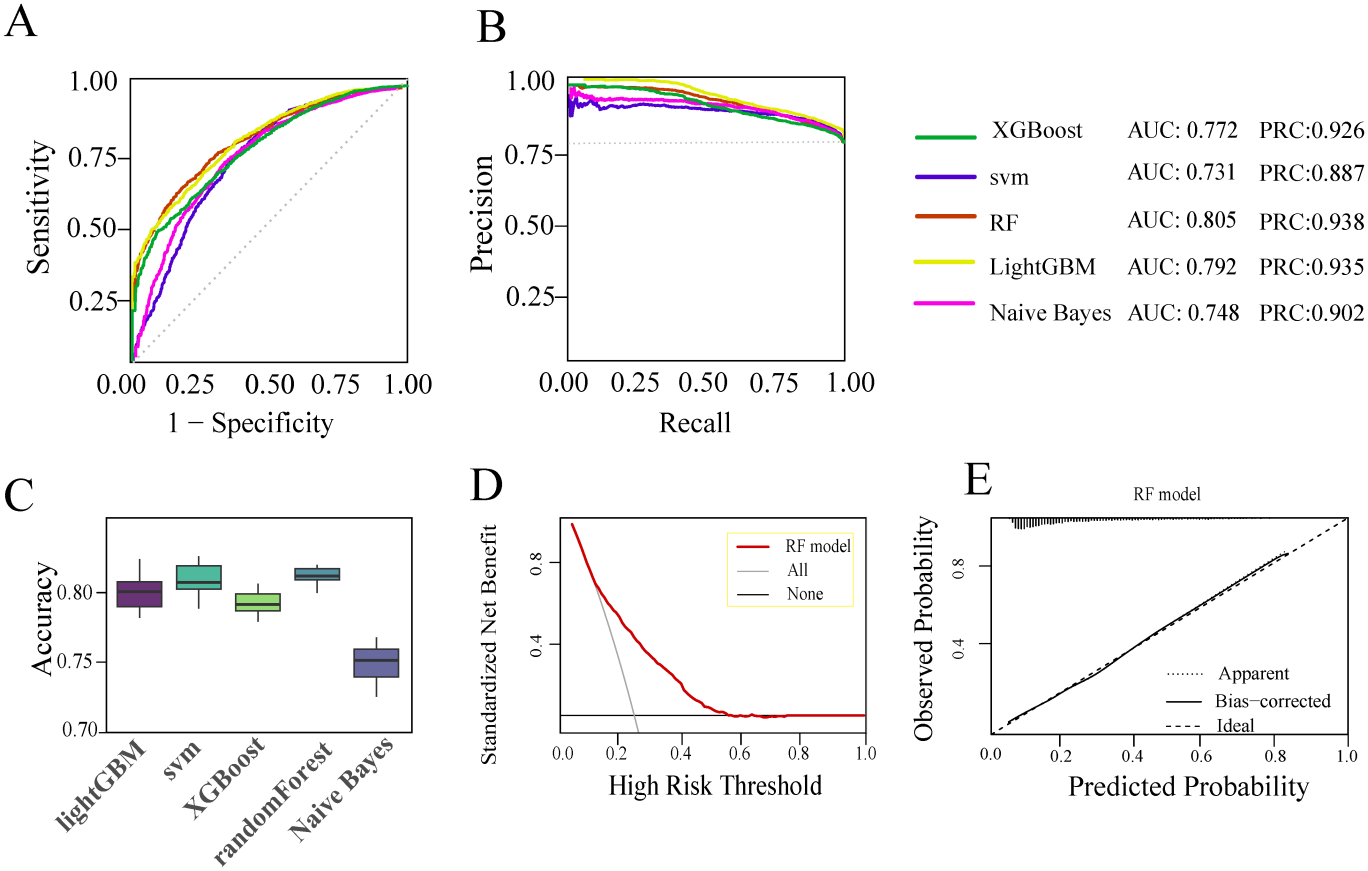
Five machine learning models for internal datasets. **(A)** ROC curves for five machine learning models. **(B)** PR curves for five machine learning models. **(C)** ACC box plots for five machine learning model. **(D)** Clinical decision curve for the RF model. **(E)** Calibration curve for the RF model.

Five machine learning algorithms were utilized to develop predictive models. As shown in **Figure 3**, the RF model demonstrated the best performance (**Figure 3A**), with an AUC of 0.80, outperforming the XGBoost (AUC = 0.77), SVM (AUC = 0.73), LightGBM (AUC = 0.79), and Naive Bayes (AUC = 0.75) models. The RF model also achieved a ROC curve AUC of 0.938 (**Figure 3B**) and the ACC of 0.82 (**Figure 3C**). The decision curve analysis indicated that the RF model provides a favorable net clinical benefit for patient screening and diagnosis (**Figure 3D**), while the calibration curve demonstrated strong agreement between predicted and observed risks (**Figure 3E**). Based on the overall performance, the RF model was selected for external validation and further evaluation. In the internal test set, the model achieved PPV(precision) of 0.83 (95%CI 0.82-0.84), TPR(Sensitivity) of 0.97 (95%CI 0.96-0.98), TNR(Specificity) of 0.26 (95%CI 0.24-0.28), negative predictive value (NPV) of 0.68, and an F1 score of 0.89 (**Table 2**).

**Table 2:** ROC curves of learning models for predicting the osteoporosis in elderly women in the test set.

Model	AUC (95% CI)	PPV(Precision) (95% CI)	TPR(Sensitivity) (95% CI)	TNR(Specificity) (95% CI)	NPV	F1 Score
XGBoost	0.77(0.75–0.79)	0.84(0.83–0.85)	0.92(0.90–0.94)	0.36(0.34–0.38)	0.54	0.88
SVM	0.73(0.71–0.75)	0.82(0.81–0.83)	0.97(0.96–0.98)	0.21(0.20–0.22)	0.69	0.89
RF	0.80(0.78–0.82)	0.83(0.82–0.84)	0.97(0.96–0.98)	0.26(0.24–0.28)	0.68	0.89
LightGBM	0.79(0.77–0.81)	0.83(0.82–0.84)	0.94(0.92–0.95)	0.31(0.29–0.33)	0.59	0.88
Naive Bayes	0.75(0.73–0.77)	0.87(0.85–0.88)	0.80(0.77–0.83)	0.56(0.54–0.58)	0.43	0.83

XGBoost, extreme gradient boosting; SVM, support vector machine; RF, random Forest; LightGBM, Light Gradient Boosting Machine; AUC, area under the ROC curve; ACC, accuracy; TPR, true positive rate; TNR, true negative rate; PPV, positive predictive value; NPV, negative predictive value.

### 3.5 Model selection and hyper-parameter tuning

We performed stratified 10-fold cross-validation on the training set with a random search over mtry, max. depth, min. node. size, num. trees, and sample. fraction. Model selection was guided by the mean cross-validated ROC-AUC (primary metric), given its threshold-independence and robustness to our ~4:1 outcome ratio; PR curve, PR-AUC, F1 Score were examined as sensitivity metrics and showed consistent rankings. The RF model was chosen as the final model. The selected configuration was max. depth=29, min. node. size=11, mtry =2, num. trees=275, sample. fraction=0.325.

### 3.6 External validation of model characteristics

**Figure 4:**
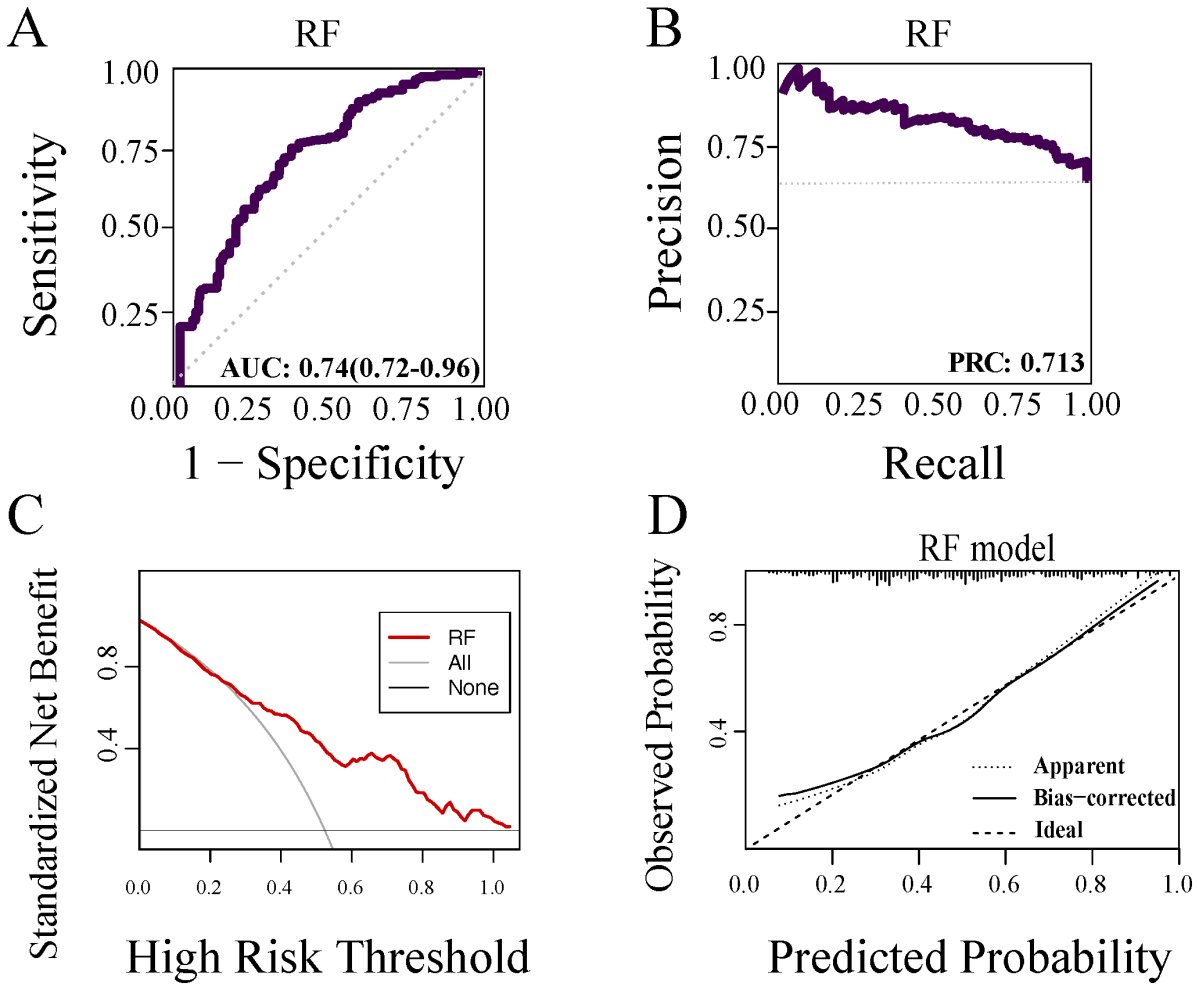
RF machine learning models for external data. **(A)** ROC curve of the external data RF model. **(B)** PR curve of the RF model. **(C)** Clinical decision curve of the RF model. **(D)** Calibration curve of the RF model.

Using data from 338 patients at Nanjing Drum Tower Hospital as an external validation cohort, as shown in **Figure 4**, the RF model constructed from the ten identified risk factors yielded an area under the curve (AUC) of 0.74 (95% CI 0.72–0.76, **Figure 4A**) with an accuracy of 0.66 (95% CI 0.65–0.67), a sensitivity of 0.72 (95% CI 0.70–0.74), a specificity of 0.66 (95% CI 0.64–0.68). The PR curve, Clinical decision curve, and calibration curve (**Figures 4B–D**) further supported the model’s favorable generalizability and clinical applicability.

### 3.7 Interpretability analysis

**Figure 5:**
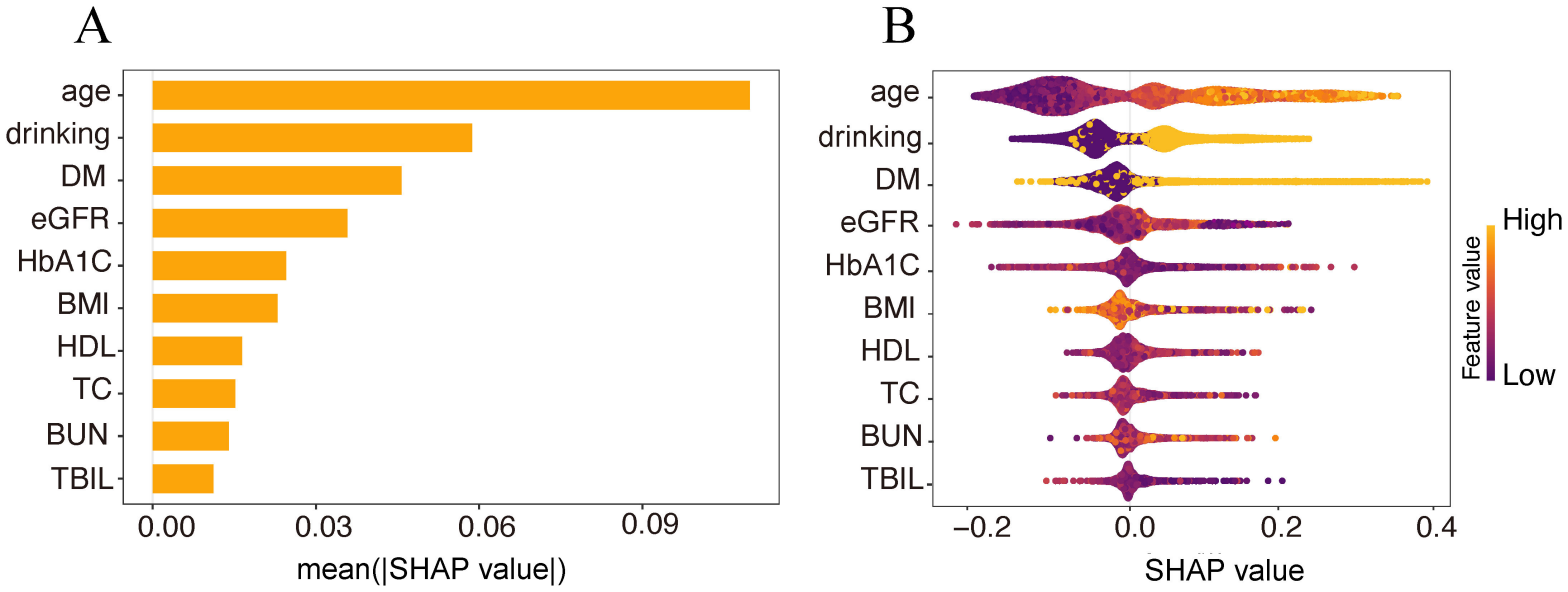
SHAP explanatory model. **(A)** SHAP explanation variable importance ranking bar chart. **(B)** variable feature change honeycomb chart.

The SHAP method was further applied to evaluate the importance of each feature variable in the RF model and their respective contributions to the model’s predictions. The visualization results indicated the following ranking of variable importance: age, drinking, DM, eGFR, HbA1c, BMI, HDL, TC, BUN, and TBIL. The bar plot illustrates the relative importance of each variable and its overall contribution to the model predictions (**Figure 5A**). The SHAP summary (bee swarm) plot (**Figure 5B**) depicts the direction and magnitude of each variable’s effect across the dataset: yellow represents higher values, purple represents lower values, with points distributed to the left indicating a negative association with osteoporosis risk, and those to the right indicating a positive association.

## 4 Discussion

Machine learning, a branch of artificial intelligence, provides substantial advantages for clinical prediction due to its ability to identify intricate and nonlinear relationships among variables, often surpassing the capabilities of traditional statistical models (11). In this study, five ML algorithms were developed to predict osteoporosis risk in elderly women using both public and real-world datasets. Among them, the RF model showed superior discrimination, calibration, and clinical utility, suggesting its potential as a tool for early screening and targeted intervention.

Traditional osteoporosis screening tools have been developed to identify individuals at increased risk of osteoporosis, including the Fracture Risk Assessment Tool (FRAX), Osteoporosis Risk Assessment Instrument (ORAI), and Osteoporosis Risk Index (OSIRIS), which are widely used due to their simplicity and clinical applicability (12). However, their dependence on predefined input variables and assumptions specific to certain populations restricts their applicability and generalizability. In contrast, ML approaches incorporate diverse demographic, clinical, and biochemical data, offering individualized risk stratification. By leveraging hospital data from elderly Chinese women, our model enhances relevance to this specific population. Shim et al. (13) and Suh B et al. (14) have reported comparable predictive accuracy (AUROC >0.74), reinforcing the feasibility of ML in osteoporosis risk modeling. Nevertheless, they did not include external validation using an independent, real-world hospital dataset. Our approach depends solely on routine laboratory measurements and basic medical history and does not depend on DXA, imaging modalities, or complex questionnaires. Leverage SHAP-based feature attribution to yield clinically interpretable, actionable recommendations.

LASSO regression, a regularization method effective in high-dimensional settings with multicollinearity, identified ten key predictors for model construction (15). In previous studies, variables such as drinking and diabetes were rarely included. The random forest risk prediction model developed in this study performed better, with an AUC of 0.805 for the internal dataset and an AUC of 0.74 for the external validation cohort. Its strong performance likely stems from its ensemble structure and resistance to overfitting. Zhang Y et al. (16) developed and validated a predictive RF model for acute kidney injury in hospitalized patients, demonstrating its effectiveness in clinical risk prediction.

In our internal test set, the random forest model demonstrated high discriminative ability (AUC = 0.80), extremely high sensitivity (TPR = 0.97), and relatively high positive predictive value (0.83), but low specificity (TNR = 0.26). This characteristic reflects the model’s deliberate design strategy of operating at a high sensitivity threshold to minimize misdiagnosis of osteoporosis. This model is intended as an adjunct rather than an independent diagnostic rule. The distribution of results in this cohort (approximately 21% osteoporosis prevalence; positive-to-negative ratio of approximately 1:4) aligns with real-world osteoporosis screening populations (17, 18). This model is explicitly positioned as a screening tool to identify high-risk individuals requiring further evaluation and cannot replace DXA. Looking ahead, we recognize that the balance between sensitivity and specificity depends on threshold settings and specific contexts: while raising decision thresholds may improve specificity and reduce unnecessary DXA referrals, it may also lead to decreased sensitivity.

SHAP analysis revealed that age was the most influential predictor, consistent with known mechanisms of age-related bone loss, including reduced osteoblast activity, increased bone resorption, and postmenopausal estrogen deficiency (19). We observed that the average age of individuals in the osteoporosis group was higher than that in the non-osteoporosis group in both the internal and external datasets. These findings highlight the need to prioritize early screening and preventive measures for the elderly population.

Participants with osteoporosis were significantly older, underscoring the importance of age targeted screening strategies. Diabetes mellitus was another key predictor (20). Chronic hyperglycemia and insulin resistance may impair bone formation by promoting oxidative stress, decreasing osteoblast activity, and increasing the accumulation of advanced glycation end products, ultimately compromising bone integrity (21, 22). Interestingly, the non-osteoporosis group had slightly higher HbA1c levels, possibly reflecting the protective mechanical and hormonal effects of obesity. This finding highlights the need to consider the broader metabolic context when evaluating glycemic status and bone health.

Alcohol use and low BMI were also significantly associated with osteoporosis (23). Chronic alcohol intake disrupts calcium homeostasis, suppresses testosterone, and promotes osteoclastic activity, all contributing to bone loss (24). Low BMI may reflect malnutrition and reduced skeletal loading, both critical factors in bone mass maintenance (25, 26). Several biochemical markers were also independently associated with osteoporosis. TC and eGFR, along with higher levels of HDL, BUN, and TBIL, emerged as significant predictors. Low TC may reflect underlying nutritional deficiencies, whereas elevated HDL, despite being beneficial for cardiovascular health, has demonstrated inconsistent associations with bone health outcomes (27). In the SHAP analysis, HDL-C and TBIL emerged as non-traditional but informative biochemical predictors of osteoporosis. Higher HDL-C values clustered on the positive side of the SHAP axis, indicating that, within the range observed in our data, elevated HDL-C contributed to increased predicted osteoporosis risk. Although HDL-C is generally considered protective for cardiovascular disease, epidemiologic findings regarding bone health have been inconsistent; notably, Li et al. reported that higher serum HDL-C was associated with increased osteoporosis risk among 790 postmenopausal Chinese women, which is concordant with our SHAP-based interpretation (28). TBIL, an endogenous antioxidant, showed a modest positive contribution to osteoporosis risk, supporting the concept of a biphasic effect whereby both very low and relatively high bilirubin concentrations may impair osteoblast function and disturb bone remodeling (29). Bilirubin levels often reflect the body’s oxidative stress burden and underlying hepatic function status. Mildly elevated concentrations may partially mitigate inflammation and oxidative damage, whereas marked abnormalities may, through mechanisms such as cholestasis, impaired absorption of fat-soluble vitamins, and pro-inflammatory responses, accelerate bone loss and alter bone remodeling dynamics. Previous population studies have also suggested that TBIL may exhibit a U-shaped or J-shaped relationship with bone mineral density, and the risk thresholds may differ across sex subgroups and according to baseline liver disease status. Therefore, we currently regard TBIL as an integrated marker of metabolic and oxidative stress, and its causal relationship with osteoporosis, as well as the underlying biological mechanisms, warrants further clarification in prospective cohorts and mechanistic experimental studies. Importantly, when we examined these variables by cohort, the direction of association for HDL-C and TBIL was similar in both the NHANES and Chinese hospital datasets. The relationship between TBIL and osteoporosis may be nonlinear and modulated by factors such as gender, age, liver function status, and other metabolic conditions. Renal function markers such as BUN and eGFR reflect bone-kidney interactions, particularly in calcium-phosphate metabolism and vitamin D homeostasis among older adults (30, 31).

Although the model showed good performance, several limitations should be noted. First, the retrospective design does not allow causal inference between predictors and osteoporosis. Second, although we performed external validation, the external cohort was relatively small, which may limit generalizability. Future work should include larger, prospective, multi-center cohorts to further optimize and calibrate the model for clinical use. Finally, we focused on postmenopausal women because they are at highest fracture risk due to estrogen deficiency, whereas male osteoporosis is less common and often secondary to heterogeneous causes (e.g., hypogonadism, glucocorticoid use, alcohol, comorbidities), implying different risk structures and intervention thresholds. A dedicated model for men will require targeted sampling and external validation in future studies. Additionally, our model did not include omics derived predictors, such as polygenic risk scores or other genomics and transcriptomics-based features, which could further improve discrimination and enhance transportability in future work.

We acknowledge that the external validation cohort is relatively small, and the distributional differences between the internal and external cohorts may affect the model’s transferability. Although domain adaptation or recalibration techniques were not applied in the current study, we plan to explore these strategies in future research to improve model transferability across diverse populations.

## 5 Conclusion

This study identified key predictive variables using public database and developed a machine learning based prediction model. The model integrates easily obtainable demographic, lifestyle, and laboratory data, offering a practical and interpretable tool for individualized risk stratification. Its robust performance in both internal and external datasets highlights its potential to enhance early detection and guide preventive care. Combining SHAP explain ability methods to interpret the intrinsic information from RF model may prove clinically useful and help clinicians tailor precise management, which is crucial for maximizing prevention and treatment in patients with early-stage osteoporosis.

## Data Availability

The original contributions presented in the study are included in the article/**supplementary material**. Further inquiries can be directed to the corresponding author.

## Supplementary material

Supplementary Figure 1 Correlation heat map of the 10 LASSO-selected predictors (Pearson/Spearman r).
