# Supplementary material for: Development and external validation of models to improve prediction of osteoporosis in elderly women: interpretable machine learning
Source: Front Endocrinol (Lausanne). 2026 Jan 9;16:1719698. doi: 10.3389/fendo.2025.1719698 (PMC12827086; doi:10.3389/fendo.2025.1719698)
Supplement: Supplementary file 2 [file Table1.docx]

Supplementary Table S1. Cross-cohort Harmonization of Laboratory & Definitions for analytes & definitions (NHANES and Chinese hospital)

| variable | NHANES variable(file) | NHANES unit (release) | NHANES platform /method | NHANES standardization/calibration | **Institutional analyzer** in Drum Tower Hospital | Institutional unit in Drum Tower Hospital | Harmonized unit (target) |
| --- | --- | --- | --- | --- | --- | --- | --- |
| Age | RIDAGEYR (DEMO) | years | Household interview / MEC exam |  | **medical history inquiry** | years | years |
| BMI | BMXBMI (BMX) | kg/m² | MEC exam | QA/QC | **Anthropometric measurements** | kg/m² | kg/m² |
| WBC | LBXWBCSI (CBC) | 10³/µL | Beckman Coulter UniCel DxH 800/880 | Routine quality control (QC) per laboratory policy | Sysmex XE-5000 Automated Hem atology System | 10⁹/L | 10⁹/L  （1×10³/µL=1×10⁹/L） |
| RBC | LBXRBCSI (CBC) | 10⁶/µL | Beckman Coulter UniCel DxH 800/880 | Routine quality control (QC) per laboratory policy | Sysmex XE-5000 Automated Hematology System | 10¹²/L | 10¹²/L  （1×10^6^/µL=1×10^12^/L） |
| Hemoglobin | LBXHGB (CBC) | g/dL | Beckman Coulter UniCel DxH 800/880 | Routine quality control (QC) per laboratory policy | Sysmex XE-5000 Automated Hematology System | g/L | g/L  （1g/dL=10g/L） |
| Platelet | LBXPLTSI (CBC) | 10³/µL | Beckman Coulter UniCel DxH 800/880 | Routine quality control (QC) per laboratory policy | Sysmex XE-5000 Automated Hematology System | 10⁹/L | 10⁹/L  （1×10^3^/µL=1×10^9^/L） |
| ALT | LBXSATSI (BIOPRO) | U/L | Roche Cobas 6000/8000（c501）, enzymatic rate method | Two-point calibration with Roche C.f.a.s. (Calibrator for Automated Systems) | BECKMAN COULTER AU5800, Lactate dehydrogenase (LDH) method | U/L | U/L |
| AST | LBXSASSI (BIOPRO) | U/L | Roche Cobas（c501）, enzymatic rate method | Two-point calibration with Roche C.f.a.s. | BECKMAN COULTER AU5800，MDH method | U/L | U/L |
| GGT | LBXSGTSI (BIOPRO) | U/L | Roche Cobas（c501）, enzymatic rate method | Two-point calibration with Roche C.f.a.s. | BECKMAN COULTER AU5800，enzymatic rate method | U/L | U/L |
| Albumin | LBDSALSI (BIOPRO) | g/L | Roche Cobas（c501）, BCP method | traceable to reference materials | Suzhou ChonTech BioPharma，bromocresol green (BCG) colorimetric method | g/L | g/L |
| Serum creatinine | LBXSCR；LBDSCRSI | µmol/L | Roche Cobas enzymatic assay (IDMS-traceable) | traceable to reference materials | Sarcosine oxidase method | µmol/L | µmol/L |

Note: eGFR was calculated from **standardized** serum creatinine (Scr).

Supplementary Table S1 (continued). Cross-cohort Harmonization of Laboratory & Definitions for analytes & definitions (NHANES and Chinese hospital)

| variable | NHANES variable(file) | NHANES unit (release) | NHANES platform /method | NHANES standardization/calibration | **Institutional analyzer** in Drum Tower Hospital | Institutional unit in Drum Tower Hospital | Harmonized unit (target) |
| --- | --- | --- | --- | --- | --- | --- | --- |
| Total bilirubin | LBDSTBSI（µmol/L） | µmol/L | Roche Cobas（c501）, Diazo-coupling colorimetric method using dichlorobenzene diazonium salt | standardized against the Doumas reference method. | Diazo method | µmol/L | µmol/L |
| BUN | LBXUN (BIOPRO) | mg/dL | Roche Cobas, Urease–GLDH kinetic UV method | Routine quality control (QC) per laboratory policy | Urease–glutamate dehydrogenase (GLDH) method | mmol/L | mmol/L  [Urea(mmol/L)=BUN(mg/dL)×0.357] |
| FBG | LBDGLUSI | mmol/L | Roche Cobas Hexokinase | no additional calibration | Glucose oxidase method | mmol/L | mmol/L |
| HbA1c | LBXGH (GHB) | % | HPLC（Tosoh G8 / Bio-Rad D-100） | no correction required | BIO-RAD, High-performance liquid chromatography (HPLC) | % | % |
| Total cholesterol | LBXTC (TCHOL) | mg/dL | Roche Cobas, CHOD–PAP enzymatic method | participates in CDC/CAP proficiency testing (PT) programs. | CHOD–PAP enzymatic method | mmol/L | mmol/L  [TC(mmol/L)=TC(mg/dL)×0.02586] |
| HDL-C | LBDHDD (HDL) | mg/dL | Roche Cobas, direct homogeneous method | participates in CDC/CAP proficiency testing (PT) programs. | direct homogeneous method | mmol/L | mmol/L  HDL-C(mmol/L)=HDL-C(mg/dL)×0.02586 |
| Triglycerides | LBXTR (TRIGLY) | mg/dL | Roche Cobas, GPO-PAP | participates in CDC/CAP proficiency testing (PT) programs. | GPO-POD method | mmol/L | mmol/L  [TG (mmol/L)=TG (mg/dL)×0.01129] |
| eGFR |  | mL/min/1.73m² | CKD-EPI 2021 | using the race-free 2021 CKD-EPI equation | CKD-EPI 2009 | mL/min/1.73m² | mL/min/1.73m² |

Supplementary Table S1 (continued). Cross-cohort Harmonization of Laboratory & Definitions for analytes & definitions (NHANES and Chinese hospital)

| variable | NHANES variable(file) | NHANES unit (release) | NHANES platform /method | NHANES standardization/calibration | **Institutional analyzer** in Drum Tower Hospital | Institutional unit in Drum Tower Hospital | Harmonized unit (target) |
| --- | --- | --- | --- | --- | --- | --- | --- |
| Alcohol consumption | ALQ110, ALQ101, ALQ130 | Questionnaire (self-report) | Interviewer-administered household interview | / | **medical history inquiry** | / | YES/NO |
| Diabetes (DM) | DIQ010, DIQ050, DIQ070 | Questionnaire (self-report) ± laboratory assays | Household interview ± laboratory assays | / | medical history inquiry± laboratory assays | / | YES/NO |
| Hypertension (HTN) | BPQ020, BPQ050A,BPQ150 | Questionnaire (self-report) ± physical examination | Household interview ± MEC blood pressure measurement | / | medical history inquiry± laboratory assays | / | YES/NO |

Note: “±” indicates that the item may or may not be included.

**Diabetes mellitus.** Questionnaire (NHANES DIQ module). Diabetes was classified if any of the following held:

1.Self-reported diagnosis — DIQ010 = “Yes” to: “*Other than during pregnancy, have you ever been told by a doctor or health professional that you have diabetes or sugar diabetes?*”

2.FPG ≥ 126 mg/dL; or

3.HbA1c ≥ 6.5%; or

4.Current medication use — DIQ050/DIQ070 = “Yes” to:

• DIQ050: “*Are you now taking insulin?*”

• DIQ070: “*Are you now taking diabetic pills to lower your blood sugar? These are sometimes called oral agents or oral hypoglycemic agents.*”

The numeric thresholds follow ADA diagnostic criteria (A1C ≥6.5% or FPG ≥126 mg/dL).

If distinguishing diagnosed vs. undiagnosed diabetes, CDC/NCHS defines undiagnosed diabetes as no self-reported diagnosis and FPG ≥126 mg/dL or A1C ≥6.5% (NHANES surveillance methods).

**External Chinese hospital cohort**: Diabetes diagnosis followed the same ADA criteria (HbA1c ≥ 6.5%, FPG ≥ 126 mg/dL(≥**7.0 mmol/L**）, physician diagnosis, or current glucose-lowering therapy).

# Alcohol use (Drinking). Data source & variables (NHANES Alcohol Use Questionnaire, ALQ module)

# ALQ110 — Lifetime drinking (≥12 drinks, ever). *“In {your/SP’s} entire life, {have you/has he/has she} had at least 12 drinks of any type of alcoholic beverage?”*

ALQ101 — ≥12 drinks in any one year.
*“In any one year, {have you/has SP} had at least 12 drinks of any type of alcoholic beverage? By a drink, I mean a 12 oz. beer, a 5 oz. glass of wine, or a one and a half ounces of liquor.”*

ALQ120Q / ALQ120U — Drinking frequency in the past 12 months.
*ALQ120Q:* *“In the past 12 months, how often did {you/SP} drink any type of alcoholic beverage? PROBE: How many days per week, per month, or per year did {you/SP} drink?”* (Instruction: *ENTER QUANTITY. ENTER ‘0’ FOR NEVER.*)
*ALQ120U:* *“UNIT OF MEASURE.”* (values: week / month / year).

ALQ130 — Average drinks per drinking day (past 12 months).
*“In the past 12 months, on those days that {you/SP} drank alcoholic beverages, on the average, how many drinks did {you/he/she} have? By a drink, I mean a 12 oz. beer, a 5 oz. glass of wine, or one and a half ounces of liquor.”*

The questionnaire text explicitly defines “1 drink = 12 oz beer / 5 oz wine / 1.5 oz liquor.” In U.S. terms, one standard drink contains ~14 g of pure alcohol.

**Drinking status was defined by ALQ101 (≥12 drinks in any one year) in our study.**

**Hypertension**

Examination: Cuff-based blood pressure measurement; the mean of repeated readings was used (BPXSY1–BPXSY3; BPXDI1–BPXDI3). NHANES transitioned from mercury sphygmomanometers to mercury-free devices, with procedures and quality control following AHA-recommended protocols.

Questionnaire: Ever told had hypertension (BPQ020); currently taking antihypertensive medication (BPQ050A for 2003–2020; changed to BPQ150 for 2021–2023).Definition: Hypertension was determined using the mean of multiple BP readings combined with medication information. In the primary analysis, we applied the 2017 ACC/AHA framework: SBP ≥140 mmHg or DBP ≥90 mmHg, or current antihypertensive treatment (BPQ050A/BPQ150).
