# Supplementary material for: Development and external validation of models to improve prediction of osteoporosis in elderly women: interpretable machine learning
Source: Front Endocrinol (Lausanne). 2026 Jan 9;16:1719698. doi: 10.3389/fendo.2025.1719698 (PMC12827086; doi:10.3389/fendo.2025.1719698)
Supplement: Supplementary file 3 [file Table2.docx]

**Supplementary Table S2.** Evaluation of Traditional Regression Prediction Models for NHANES Cohort Subgroups

|  | AUC(95%CI) | Sensitivity | Specificity |
| --- | --- | --- | --- |
| subgroup |  |  |  |
| Mexican American | 0.748(0.691,0.805) | 0.841 | 0.565 |
| Other Hispanic | 0.713(0.630,0.796) | 0.805 | 0.593 |
| Non-Hispanic White | 0.734(0.710,0.758) | 0.722 | 0.562 |
| Non-Hispanic Black | 0.740(0.675,0.805) | 0.731 | 0.689 |
| Other Race-Including Multi-Racial | 0.777(0.702,0.851) | 0.837 | 0.63 |
